# Management and Prevention of Cellular-Therapy-Related Toxicity: Early and Late Complications

**DOI:** 10.3390/curroncol30050378

**Published:** 2023-05-15

**Authors:** Simon R. Mucha, Prabalini Rajendram

**Affiliations:** 1 Department of Critical Care Medicine, Respiratory Institute, Cleveland Clinic, Cleveland, OH 44195, USA; muchas@ccf.org; 2 Critical Care Medicine, Department of Anesthesiology and Critical Care Medicine, Memorial Sloan Kettering Cancer Center, New York, NY 10065, USA

**Keywords:** chimeric antigen receptor T-cell therapy (CAR-T), adoptive immune cell therapy, cytokine release syndrome, late complications, cytopenias, hypogammaglobulinemia, immune effector cell-associated neurotoxicity syndrome (ICANS), intensive care unit (ICU))

## Abstract

Chimeric Antigen Receptor T (CAR-T) cell therapy has dramatically changed prognosis and treatment of relapsed and refractory hematologic malignancies. Currently the 6 FDA approved products target various surface antigens. While CAR-T therapy achieves good response, life-threatening toxicities have been reported. Mechanistically, can be divided into two categories: (1) toxicities related to T-cell activation and release of high levels of cytokines: or (2) toxicities resulting from interaction between CAR and CAR targeted antigen expressed on non-malignant cells (i.e., on-target, off-tumor effects). Variations in conditioning therapies, co-stimulatory domains, CAR T-cell dose and anti-cytokine administration, pose a challenge in distinguishing cytokine mediated related toxicities from on-target, off-tumor toxicities. Timing, frequency, severity, as well as optimal management of CAR T-cell-related toxicities vary significantly between products and are likely to change as newer therapies become available. Currently the FDA approved CARs are targeted towards the B-cell malignancies however the future holds promise of expanding the target to solid tumor malignancies. Further highlighting the importance of early recognition and intervention for early and late onset CAR-T related toxicity. This contemporary review aims to describe presentation, grading and management of commonly encountered toxicities, short- and long-term complications, discuss preventive strategies and resource utilization.

## 1. Introduction

Chimeric Antigen Receptor T (CAR-T) cell therapy has dramatically changed the prognosis and treatment of relapsed and refractory hematologic malignancies. Currently there are 6 CAR-T products with U.S food and drug administration (FDA) approval for the treatment of specific subtypes of B-cell non-Hodgkin lymphoma (NHL), adult and pediatric B-cell acute lymphoblastic leukemia (ALL) and multiple myeloma (MM), with many more currently under investigation for various other hematologic and solid tumor malignancies [1], and even autoimmune and degenerative diseases [2,3,4].

CAR-T cells are genetically modified T-lymphocytes expressing a chimeric antigen receptor (CAR) designed to target and kill cancer cells expressing certain surface antigens. This adaptive “living drug therapy” combines the specificity of a targeted antibody with the cytotoxic and memory functionality of T-cells to induce a potent and durable anti-tumor response. While CAR-T therapy can achieve high response rates (ZUMA-1 trial: complete response (CR) of 58% [5] and dramatically improve survival (ZUMA-1 5 year survival of 42.6% and 63% in patients with CR) [6] in heavily pre-treated, refectory and relapsed disease it is also associated with unique, severe and potentially life-threatening toxicity that requires specialized expertise in management.

Cytokine Release Syndrome (CRS) and Immune Effector Cell-Associated Neurotoxicity Syndrome (ICANS) are unique to CAR-T cell therapy and clearly distinct from other autoimmune toxicity [5]. As CAR-T therapy is administered more routinely for refractory/relapsed disease, it is essential for all providers caring for patients with B-cell malignancies to become familiar with the manifestation and management of its unique toxicities. The following paper aims to provide a contemporary review of the presentation, grading and management of commonly encountered toxicities associated with commercially available CAR-T therapies, according to the most recent guideline recommendations by the American Society of Clinical Oncology (ASCO) [7], National Comprehensive Cancer Network (NCCN), and the American Society for Transplantation and Cellular Therapy (ASTCT) [8,9]. Where appropriate, we will describe preventive strategies for short- and long-term complications, discuss resource utility in the management of toxicities/complications and finally briefly review emerging effector cell therapies.

## 2. Chimeric Antigen Receptor (CAR) Products

**Figure 1:**
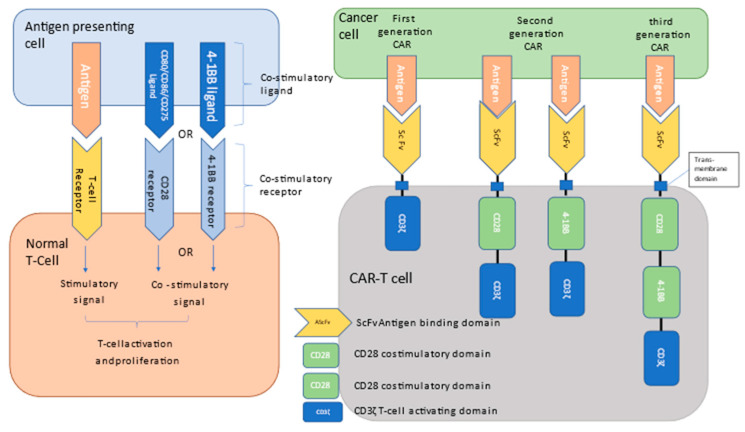
CAR constructs and mechanism of action. T-cell activation by antigen-presenting cells requires both T-cell receptor binding of the target antigen as well as a simultaneous co-stimulatory signal from either the CD28 or 4-1BB receptor to achieve full activation and proliferation. CAR-T cells utilize an ScFv-Antigen binding domain linked to the intracellular activation domain (CD3ζ). First, general CAR-T produces laced CD28 or 4-1BB costimulatory domains and fails to fully activate and promote T-cell proliferation. Currently commercially available 2nd-generation CAR-T constructs utilize either DC28 or 4-1BB costimulatory domains. #rd generation constructs, combining both CD28 and 4-1BB domains, aim to harness the relative advantage of both to minimize toxicity and maximize efficacy through robust activation, proliferation, and persistence.

All CARs are synthetic constructs that combine an extracellular immunoglobulin-derived singe-chain variable fragment (scFv) designed to bind to the target antigen, a transmembrane domain, and a T-cell activating domain (usually the Zeta chain of the CD3 complex). Currently commercially available CAR-T constructs also include a costimulatory domain, such as CD28 or 4-1BB which provides an additional signal for T-cell activation, leading to robust activation, proliferation, and antitumor activity (Figure 1) [10].

Currently, four CAR-T products targeting CD19 are FDA approved for the treatment of relapsed/refractory (R/R) B-cell lymphomas and B-cell ALL [5,11,12,13]. Two products targeting B-cell maturation antigen (BCMA) are approved for the treatment of multiple myeloma [14,15]. Clinical outcomes and rates of toxicity in pivotal trials that led to the FDA approval of the currently commercially available CAR-T products are summarized in Table 1 [8]. Since their approval, these products have rapidly been considered more routinely for the treatment of patients with R/R lymphoma, ALL and MM. Observational studies and registry data, summarized in Table 2, demonstrate striking similarity in clinical outcomes in the “real world” utilization of commercially available CAR-T therapies [16,17,18,19,20,21,22,23,24,25,26,27,28,29,30].

Regardless of the CAR-T product used, underlying cancer diagnosis, or patient-specific risk factors, toxicities occur almost universally. They can be broadly categorized as (1) systemic manifestations of a severe inflammatory response following target recognition and CAR-T cell activation (CRS, hemophagocytic lymphohistiocytosis [HLH]/macrophage activation syndrome (MAS] and CANS) [32] and (2) on-target/off-Tumor toxicity such as cytopenias and persistent B-cell aplasia leading to hypogammaglobulinemia and increased early and long term risk of infections [33]. Both require close monitoring and early targeted interventions to prevent serious and potentially fatal complications.

## 3. Chimeric Antigen Receptor-Mediated Toxicities

### 3.1. Cytokine Release Syndrome (CRS)

Cytokine release syndrome is defined as a supraphysiologic response following any immune therapy that results in the activation or engagement of endogenous or infused T cells and/or other immune effector cells (e.g., lymphocytes, myeloid cells) [34]. Symptoms can be progressive; characteristically include fever at the onset; and may include hypotension, capillary leak (hypoxia from pulmonary edema) and end-organ dysfunction [35]. Various grading systems have been used in the past to quantify the severity of CRS; however, to standardize reporting and management of CRS, the ASTCT consensus criteria were developed in 2019. The ASTCT criteria defined CRS according to the presence of fever (temperature ≥ 38 °C, not attributable to another cause) within the expected timeframe of initiation of treatment and graded the severity according to the degree of hypotension and hypoxia (Table 3).

The pathophysiology of CRS is thought to be the sudden release of inflammatory cytokines in response to CAR-T cell and immune effector cell activation, leading to endothelial activation and injury, capillary leak, coagulopathy, cardiopulmonary instability and multi-system organ dysfunction [32,36]. Multiple cytokines and signaling pathways have been implicated in the pathogenesis and progression of CRS including Interleukin (IL)6; IL1; Interferon gamma (IFN-g); and Tumor Necrosis Factor-alpha (TNF-α), with IL6, IL1, and nitric oxide (NO) derived from activated macrophages being considered the central mediators of severe presentations [36,37,38,39]. Elevation of certain cytokines within 36 h of CAR-T cell administration has been shown to be associated with a high risk of severe (≥grade 4) CRS.

The typical time to onset for CRS is 2 to 3 days, with a duration of 7 to 8 days, although CRS may occur within hours and as late as 10 to 15 days after CAR T-cell infusion. Risk factors for CRS vary between studies and include high tumor burden (or elevated lactate dehydrogenase [LDH] as a surrogate marker thereof) [23], conditioning regiment, dose and type of CAR-T cell product infused, as well as elevated biomarkers of inflammation (C-reactive protein [CRP], IL6 levels, Ferritin) and endothelial activation prior to and in response to therapy [40,41]. 

Anti-inflammatory therapy, specifically targeting IL6, has become the cornerstone of CRS management. Tocilizumab, a humanized IgG1k anti-IL-6R antibody binds to both soluble and membrane-bound IL-6R, blocking the downstream signal transduction pathways implicated in CRS. It is currently the only anti-IL6 therapy approved by the FDA for the treatment of severe or life-threatening CAR T cell–induced CRS [42]. While it is approved for severe or life-threatening CRS, current guidelines and product information recommend the initiation of tocilizumab for the treatment of grade ≥ 2 or grade 1 CRS in patients at high risk of early and severe CRS or those whose symptoms persist greater than 24 h. For severe (grade ≥ 3) or refractory CRS the addition of steroids is recommended [7,43,44]. A recent subgroup analysis of the ZUMA-1 study of axicabtagene-ciloleucel (axi-cel), showed 95% and 80% objective and complete response rates, respectively, for patients who received prophylactic steroids (dexamethasone 10 mg on day 0 (pre-infusion), day 1 and 2) or early addition of steroids to tocilizumab for CRS [45], challenging the theoretical concerns that steroids could reduce efficacy and affect the durable response and CR. 

Persistent or refractory CRS, not responding to two to three doses of tocilizumab may necessitate alternative strategies. Alternative agents targeting the IL6 or IL1 pathways implicated in CRS include siltuximab (monoclonal antibody against IL6), ruxolitinib (a Janus kinase inhibitor that inhibits downstream IL6 signaling), and the IL1 receptor antagonist, anakinra. These agents are increasingly incorporated into the routine treatment of severe or refractory CRS/ICANS [46,47], but are considered rescue treatment in current guidelines, citing limited anecdotal evidence and the need for validation in ongoing prospective studies [7,43,44].

### 3.2. Hemophagocytic Lymphohistiocytosis (HLH)

CRS shares many clinical features with secondary HLH (sHLH) or Macrophage Activation Syndrome (MAS), a life-threatening hyperinflammatory syndrome, characterized by unchecked activation of lymphocytes and macrophages, excessive cytokine production, extreme inflammation, and tissue injury. HLH can be precipitated by infections, malignancy, autoimmune diseases or hematopoietic stem cell transplantation (HSCT) [48,49]. Malignancy-related HLH occurs in approximately 1% of patients with hematologic malignancies [50,51,52]. HLH has been reported following CAR-T cell therapy (CAR-HLH) but may go unrecognized and underreported due to its overlapping symptoms with severe CRS [53,54]. Both the 2004 HLH diagnostic criteria and the H-score have been shown to be of low diagnostic utility in diagnosing CAR-HLH or distinguishing CAR-HLH from CRS [55]. Proposed diagnostic criteria for CAR T-cell–related HLH include rapidly rising and severely elevated ferritin of ≥5000–10,000 ng/mL, along with at least two organ toxicities, including the presence of hemophagocytosis in bone marrow or other organs, or at least grade 3 transaminitis, renal insufficiency, or pulmonary edema which typically develop after the peak of CRS (Table 4) [56].

The reported incidence of CAR-HLH in commercially available CAR-T products is between 1 and 3.5% [57,58,59]. A distinct HLH/MAS-like syndrome was described more frequently in patients treated with a CAR-T product targeting CD22 for R/R B-ALL [60,61]. In this study, CAR-HLH was associated with NK-cell lymphopenia (both pre-and persistent post-infusion), robust CART-cell and CD-8 expansion and persistently elevated HLH-associated cytokines (IFNγ and IL-1β amongst others). IL6 was elevated in those with CAR-HLH, but confounded by the administration of tocilizumab, which interestingly did not mitigate the development of CAR-HLH. In light of the profound elevations in IL1β and IFNγ, the authors credit the use of anakinra for the positive outcomes observed, where all cases of HLH resolved (with one mortality due to bacterial sepsis) and suggest considering the use of emapalumab, a monoclonal antibody targeting IFNγ. Emapalumab, is now the first FDA-approved recommendation for the treatment of refractory/progressive HLH [62]. Preclinical data suggest early emapalumab administration may impede CART- cell efficacy; thus prospective evaluations of safety and efficacy are necessary [63].

In the absence of randomized control trials (RCTs), the management of CAR-HLH is extrapolated from the immunosuppressive regimen utilized in familial and other secondary HLH. Current consensus guidelines recommend high-dose corticosteroids and IL6 antagonists and suggest consideration of anakinra in patients who show no improvement. Drawing from evidence in the treatment of bona fide HLH, ASCO and NCCN guidelines recommend the addition of etoposide for refractory cases, while the SITC recommend against it, due to etoposide’s documented toxicity to T-lymphocytes [7,43,44]. Better guidance on early recognition and optimal treatment is urgently needed, as mortality associated with CAR-HLH remains excessively high (66.9%) [59].

## 4. Immune Effector Cell-Associated Neurotoxicity Syndrome (ICANS)

Clinical manifestations of ICANS often follow a characteristic progression of symptoms evolving from mild tremor and dysgraphia to expressive aphasia (a highly specific manifestation of ICANS, documented in 88% of patients with ICANS [64]), apraxia, impaired attention followed by lethargy and depressed consciousness and in severe cases progression to stupor and coma. Seizures often occur after the development of severe (global) aphasia. In rare cases, diffuse cerebral edema may develop. The onset of neurologic symptoms occurs around 5 days (range: 2–11 days) from CAR-T cell infusion and persists for a median of 10 days (range: 1–14 days). There is a significant correlation with the presence and severity of CRS, which usually precedes any neurologic symptoms, although ICANS without CRS may occur or when CRS has resolved [65]. 

Although the exact pathophysiology of ICANS remains poorly understood, two mechanisms have been proposed. The first is cytokine-mediated toxicity whereby increased permeability of the blood-brain-barrier allows the diffusion of inflammatory elevated serum cytokines (IL6, IL10, IL15, granulocyte-macrophage colony-stimulating factor (GM-CSF), IFNγ, and TNFα) across the injured endothelium. The second is the recruitment of CAR-T and inflammatory cells into the central nervous system (CNS), both leading to microglial activation; neuroexcitatory toxicity and further inflammation, injury, and dysfunction [32,66,67]. Elevated protein levels and cerebrospinal fluid (CSF)/serum albumin ratios on CSF analysis have been associated with ICANS and may correlate with severity [65]. However, CSF analysis may be entirely normal in a majority of patients [68], and the detection of CAR-T cells within the CSF is not per se pathologic [69,70]. In contrast to other cytokines, IL8, IP10, and monocyte chemoattractant protein-1 (MCP-1) have been shown to be disproportionately elevated in the CSF of patients with severe ICANS, suggesting local production by activated myeloid, astrocyte and/or endothelial cells. Elevated CSF levels of the excitatory neurotransmitters glutamate and quinolinic acid in patients with severe ICANS may explain myoclonus and seizures observed [65]. 

A brain magnetic resonance imaging (MRI) pattern that includes reversible T2 hyperintensities in the bilateral thalami, pons, and medulla, often accompanied by symmetric white matter T2 hyperintensities that are subcortical or affect the external and extreme capsule has recently been described as characteristic for CD19 CAR-T associated ICANS. Characteristic thalamic enhancement and swelling are also present in rare cases of diffuse cerebral edema [71,72,73]. Similarly, the literature on electroencephalography (EEG) findings associated with ICANS is evolving. While most EEGs are reported as showing diffuse slowing in patients with low-grade ICANs, there is growing evidence that the degree of EEG abnormalities parallels the severity of ICANS. A pattern of generalized periodic discharges is commonly seen with higher-grade ICANS. Up to one-third of patients with severe ICANS may have evidence of seizure activity or persistent discharges at 2.5 Hz or faster (indicating non-convulsive status epilepticus [NCSE]) [64,65,74,75]. Importantly, most patients with seizures or NCSE on EEG did not demonstrate clinically obvious seizure-like activity, highlighting the importance of standardized proactive neurologic monitoring [75].

The ASTCT ICANS consensus grading scale consists of an immune effector cell-associated neurotoxicity (ICE) score, a standardized 10-point screening tool, as well as 4 neurologic domains: level of consciousness, seizure, motor findings, and elevated intracranial pressure/cerebral edema (Table 5). The overall ICANS grade is assessed using the most severe symptom in any of the five domains. Current guidelines recommend serial assessment utilizing the ASTCT ICANS scale to detect and grade ICANS. Repeat imaging and CSF evaluation are indicated if symptoms do not improve after 24 h of the initiation of treatment and EEG monitoring is recommended for all patients with grade ≥ 2 toxicity [34,44,65]. Patients with ICANS grade ≥ 2, rapidly evolving neurotoxicity and those with concurrent CRS should be considered for ICU admission to allow for close monitoring and intubation for airway protection. In addition to ICANS, patients treated with CAR-T therapy are at risk for infection and CNS hemorrhage, which may present with similar symptoms and should be ruled out at the onset of neurologic symptoms.

Steroids, the current cornerstone of treatment, are initiated and dosed based on this severity grading. Current guidelines recommend 1 dose of dexamethasone 10 mg intravenous for grade 2 ICANS, followed by repeat neurological assessment reassessment, and repeated doses every 6 to 12 h if symptoms do not improve (Table 5). ICANS commonly develops after the onset of CRS. It is important to note that tocilizumab does not cross the blood-brain-barrier and is therefore not effective in preventing or treating ICANS. Moreover, tocilizumab can lead to a transient elevation in serum IL6 levels as well as elevated levels of IL6 in the CSF, which has been linked to potentially more severe neurotoxicity. Current guidelines, therefore, recommend the addition of steroids to tocilizumab in low-grade CRS with any concurrent ICANS.

Data supporting alternative therapies for persistent or refractory ICANS are limited. Given the strong physiologic rational, the IL1 antagonist anakinra is increasingly being integrated into routine practice with promising early results [46,74,76]. Case reports also describe the use of intrathecal chemotherapy for highly refractory cases [77,78]. Although mortalities have occurred due to refractory cerebral edema, symptoms of neurotoxicity, fortunately, tend to resolve. The presence of ICANS alone does not appear to negatively affect treatment effects, such as time to progression, progression-free survival (PFS) or overall survival (OS) [64], but has been linked to infections, including late infections and infection-related mortality [23].

## 5. Toxicities and Overall Outcomes—Real World Data Explored

As compared to the patients in the pivotal trials, patients in the real-world studies were significantly older, with worse functional status and higher rates of bridge therapy [79]. Forty to sixty percent of patients treated with standard-of-care axi-cel, tisagenlecleucel (tisa-cel), or lisocabtagene-maraleucel (liso-cel) would not have met inclusion criteria for pivotal ZUMA-1, JULIET and TRANSCEND trials [24,26,80] and as high as 75–79% of patients would have been ineligible for participation in pivotal MM trials [29,30]. Remarkably, despite this older, more frail patient population, standard-of-care treatment with commercially available CAR-T is achieving essentially identical response rates [5,11,13,14,22,24,29,30] while maintaining similar or even improved rates of high-grade toxicity [5,16] (grade ≥ 3 CRS: axi-cel: 13% vs. 8% [5,16], tisa-cel: 22% vs. 9% [13,22]; brexucabtagene autoleucel (brexu-cel): 15% vs. 8% [11,29], idecabtagene vicleucel (Ide-cel) 5% vs. 4% [14,30]; grade ≥ 3 ICANS: axi-cel: 28% vs. 24% [5,16,24], tisa-cel 12% vs. 3% [13,22], ide-cel: 3% vs. 5% [14,30]). A possible explanation is the higher rate of tocilizumab and/or steroid use in the real world, which suggests that early recognition and proactive treatment may be associated with improved outcomes. 

Clinical trials also suggest earlier onset, greater severity and higher frequency of CRS (85–91% vs. 40–58%) and ICANS (63–64% vs. 21–30%) for CARs with CD28 domains (axi-cel and liso-cel) compared to those expressing 4-1BB (tisa-cel and liso-cel), respectively [5,11,12,13,16,24]. Severe toxicity was significantly less in recent trials utilizing 4-1BB costimulatory CARs ide-cel and ciltacabtagene autoleucel (cilta-cel) targeting BCMA for the treatment of multiple myeloma with rates of grade ≥ 3 CRS or ICANS observed in only 4–5% and 3–9% of patients, respectively [14,15].

In the absence of head-to-head RCTs, several recent studies aimed to compare CAR-T products in terms of efficacy and safety using various statistical methods to account for heterogeneity and confounders. Schuster at al. used a matching-adjusted indirect comparison (MAIC), assessed outcomes for tisa-cel and liso-cel by comparing 106 patients from the JULIET study to 256 patients from the TRANSCEND study and found a higher overall response rate (ORR) (72.7% vs. 62.9%), but comparable OS, PFS, and complete response rate (CRR) [81]. In a similar analysis, using summary level data from the JULIET study, Cartron et al. reported statistically significant greater response rates for liso-cel compared to tisa-celTisa-cel (ORR:OR = 2.78, 95% confidence interval (CI): 1.63–4.74; CR:OR = 2.01, 95% (CI): 1.22–3.30; PFS: hazard ratio (HR) = 0.65, 95% CI: 0.47–0.91; OS:HR = 0.67, 95% CI: 0.47–0.95) [82].

In analogous comparisons between axi-cel and liso-cel for R/R large B-cell lymphoma objective response rates were comparable, with improved OS for axi-cel (OS: HR: 0.53, 95% CI: 0.34–0.82; PFS: HR: 0.61, 95% CI: 0.40–0.92), but significantly lower odds of severe toxicity with liso-cel (CRS: OR = 0.08, 95% (CI): 0.01–0.67), neurotoxicity OR = 0.05, 95% (CI): 0.02–0.15) [83,84].

Using a directed dicyclic graph to account for confounding in a nonrandomized observational setting, Gauthier et al. compared patients treated with axi-cel, tisa-cel, or JCAR014 (a CAR design identical to liso-cel, but with differences in dosing, cell formulation, and manufacturing process). The authors found a lower risk of severe toxicity with the use of tisa-cel or JCAR014 compared to axi-cel (adjusted odds ratio (aOR) for grade ≥ 3 CRS: 0.47; 95% CI, 0.21–1.06; *p* = 0.07 and OR 0.19; 95% CI, 0.08–0.46; *p* < 0.001; and for severe ICANS ORs, 0.17; 95% CI, 0.06–0.48 and 0.06–0.47; *p* < 0.001 and *p* < 0.001). In contrast, the analysis suggests lower antitumor efficacy with tisa-cel (aOR, 0.23; 95% CI, 0.06–0.78; *p* = 0.02) and JCAR014 (aOR, 0.21; 95% CI, 0.06–0.73; *p* = 0.01) compared with axi-cel [20].

Several recent large observational and registry studies compare outcomes between the most widely used commercially available anti-CD19 CAR-T products, axi-cel and tisa-cel, in the “real world” setting [85]. The results revealed significantly higher rates of short-term toxicity after treatment with axi-cel compared to tisa-cel for advanced-stage R/R diffuse large B cell lymphomas. However, clinical outcomes were significantly better with axic-cel compared to tisa-cel, with the best ORR [22,85]. One multi-center study with 356 patients suggested a superior CR (41% vs. 31%) and 12-month PFS (35% vs. 24%) for axi-cel compared with tisa-cel, but no difference in OS was observed [23]. In contrast, in a cohort of 260 patients Riedell et al.l. reported statistically significant higher rates of toxicity with axi-cel compared to tisa-cel(grade ≥ 3 CRS: 9% vs. 1%; grade ≥ 3 ICANS: 38% vs. 1%), but no difference in ORR, CR (44 vs. 35%, *p* = 0.319), 12-month PFS (42% vs. 32%, *p* = 0.206) or OS (62% vs. 59%, *p* = 0.909) [19]. In these studies, patients receiving tisa-cel tended to be older, with more co-morbid conditions and poorer functional status than those receiving axi-cel, reflecting a common practice of avoiding the use of axi-cel in older and frail patients due to concerns about a higher risk of severe toxicity [86].

While differences in trial design, patient characteristics, toxicity grading systems and management protocols for CAR-T toxicity preclude conclusive assessment of differences between products across trials, there appear to be key differences in expansion kinetics, persistence, and toxicity between products, particularly between products utilizing CD28 or 4-1BB co-stimulatory domains [10].

## 6. On Target off Tumor Effects

Most tumor antigens targeted by CAR-T cells are not perfectly cancer-specific and therefore lead to CAR-T toxicity in other organ systems expressing the same target antigen, called on-target/off-tumor toxicity. This occurs through the engagement of target antigens in nonpathogenic tissues predictably seen in the following organ systems; gastrointestinal, pulmonary and hematologic. In the setting of CD19-specific CAR T-cells, the classic example of such on-target off-tumor toxicity is the destruction of non-malignant B-cells leading to profound and persistent B-cell aplasia and hypogammaglobulinemia. B-cell aplasia parallels the expansion of CAR-T cells, usually developing within 2 weeks of infusion, and routinely persists for over 6 months, potentially years. Indeed, the persistence of B-cell aplasia can serve as an indicator of the persistence of pharmacologically active anti-CD19 CAR-T cells [33].

The recent findings that CD19 is on brain pericytes raised the possibility of direct CD19 targeted neurotoxicity that may also contribute to CD19-associated neurotoxicity and remains to be further evaluated [87]. Movement and neurocognitive treatment-emergent adverse events (MNTs) are now considered on-target/off-tumor toxicity. MNTs are Parkinson-like symptoms characterized by and early finding of micrographia, followed by progressive bradykinesia, rigidity, gait disturbances, flat affect, personality changes, and gait and cognitive impairment following treatment with CAR-T products targeting BCMA. A possible mechanism appears to be an on-target/off-tumor effect of BCMA-targeted CAR-T cells crossing the blood–brain barrier and targeting of BCMA-expressing cells of the basal ganglia [70,88].

The severity of reported on-target/off-tumor toxicity has ranged from manageable B-cell aplasia to death resulting from severe toxicity and therefore requires early recognition and management.

## 7. Late Complications 

### 7.1. Hypogammaglobulinemia and Cytopenia

While CAR mediated-toxicities (CRS and ICANs) occur concurrently with CAR-T cell expansion and within the first 28 days of cell infusion. The most commonly reported toxicities during long-term follow-up post-CAR-T therapy are decreased B-cell counts and hypogammaglobulinemia (defined as immunoglobulin G (IgG) level of 400 mg/dL) and less common are late-onset infections, secondary malignancies, and graft vs. host disease [89,90,91]. The development of new malignancies after CAR T-cell infusion are laryngeal cancer, prostate cancer, GI stromal tumor, melanoma, hepatocellular carcinoma, and myelodysplastic syndrome (MDS) [92].

Anti-CD19 CAR T-cell therapy causes B-cell depletion and hypogammaglobulinemia. Absolute B-cell numbers recover to normal levels in 63% of patients receiving CAR T-cell, in a median time of 12 months (range 2–59 months) [92]. In the pediatric population with ALL who received tisa-cel, all patients developed B-cell aplasia and had an 83% probability of ongoing aplasia at 6 months [93], and 90% of responders required intravenous immunoglobulin (IVIG). Approximately 35% of patients have baseline hypogammaglobulinemia secondary to underlying disease or previous therapy and it has been reported that as high as 67% of adult patients with R/R B-cell NHL or CLL had hypogammaglobulinemia beyond 90 days [91]. Approximately one-third of patients receive IVIG after CAR-T cell therapy [11,13,90]. Levels of serum IgG, IgA, and IgM recover to normal level within 120 months in about 25% of patients treated with CAR-T and in the other 75% of patients, there is a persistently low level of at least 1 IG [92]. Data in primary immunodeficiency disorders suggest that hypogammaglobulinemia is associated with higher risk of infection. Therefore, as CD19 or BCMA-directed CAR-T therapy administration increases, management of hypogammaglobulinemia with an infusion of IVIG monthly to maintain a goal IgG ≥ 400mg/dL, particularly if recurrent/severe infections occur, can be considered to mitigate the risk of infections [94]. However, routine replacement has not been shown to be correlated with the incidence of infection and therefore is not supported by recent allergy and immunology guidelines, and instead, screening quantitative immunoglobulins and specific antibody titers in response to vaccines before and monthly for 6 months after initiation of CAR-T-cell therapy is suggested [95,96,97,98,99].

Prolonged cytopenia, demonstrated by hypocellularity in bone marrow biopsy, has been seen in both observational studies and clinical trials. Grade ≥ 3 cytopenias at or beyond 3 months have been reported at a rate of 17% [90] and most cytopenias resolved on long-term follow-up. In other trials, at day 28, grade ≥ 3 neutropenia and thrombocytopenia have been reported in 24% and 41% of patients, respectively [13]; the cases of neutropenia resolved on long-term follow-up (3 months), but thrombocytopenia persisted. As with hypogammaglobulinemia, the burden of grade ≥ 3 cytopenias is higher in the patients with R/R B-cell ALL compared to patients with non-Hodgkin’s lymphoma [93,100]. Numerous (>3) previous lines of therapy, baseline cytopenia, CAR construct, grade ≥ CRS/ICANS, higher peak CRP, and higher ferritin level have been associated with late cytopenias and a lower likelihood of count recovery at 1 month [100,101]. Caution should be exercised in generalizing the outcomes of the studies given the difference in CAR constructs, prior therapies administered and patient age, comorbidities, and disease burden. Management involves supportive care measures (blood product transfusion, GCSF, and prophylactic antibiotics in neutropenic patients). 

### 7.2. Late Infections-Prevention and Prophylaxis

Patients treated with CAR-T cell therapy are at significantly increased risk for infections due to various patient-, disease-, and treatment-related factors. The reported incidence of any infection in patients treated with anti-CD19 CAR-T products range from 35% to 65% within the first weeks of infusion and as high as 63% at 1 year. Grade ≥ 3 infections were reported beyond 8 weeks at a rate of 18% and from 7 to 19 months at a rate of 8% in the JULIET and ZUMA-1 trial, respectively. In this vulnerable population, infection remains the leading cause of non-relapse mortality [91,95,96,97,102].

In addition to direct cytotoxic effects, severe CRS and ICANS have been linked with persistent cytopenia and therefore risk of infection [100,103]. While there is no evidence that the use of tocilizumab is associated with increased risk of infections [104], steroid exposure has been established as a major risk factor [95,96,102,105]. The impact of the growing use of anakinra infection-risk is currently unknown [95]. Large retrospective registry studies have identified high-grade ICANS as an independent risk factor for infection and infection-related mortality [23]. 

The respiratory tract has been identified as the most common site of infection in CAR-T recipients [5,13,91]. Causative organisms have been identified as bacterial (60%), viral (31%), and fungal (9%), and approximately 80% of patients were treated in the outpatient setting and only 5% required ICU management [91]. The risk of bacterial infection most strongly correlates with the degree and duration of neutropenia. As such, most recommendations follow the guidance developed for stem cell transplant recipients and recommend antibiotic prophylaxis (using fluroquinolone or extended-spectrum beta-lactam based on local antibiogram and standard of care) for the duration of severe neutropenia. Infections occurring ≥ 6 months are rare [92]; late re-activation of herpetic and zoster infections have been reported, particularly after treatment with axi-cel, and extended duration acyclovir prophylaxis is now recommended [17,95,99]. Atypical infections such as human herpes virus-6 encephalitis and systemic mycosis have been reported in patients with ALL [93]. Similarly, pneumocystis jirovecii pneumonia prophylaxis per institutional standards is recommended for 6–12 months or until the CD4 count is >400 cells/µL. Antifungal prophylaxis should be provided for high-risk patients and those treated with tocilizumab and/or corticosteroids.

### 7.3. Early and Late Organ Support 

Cardiac- and renal-organ-specific toxicity related to CAR-T therapy has been described in the literature. Acute kidney injury (AKI) and chronic kidney disease (CKD) have been well studied in patients undergoing hematopoietic stem cell transplant (HSCT) [106,107]. AKI has been reported in 5–30% of patients post-CAR-T therapy [108,109,110]. The risk of developing AKI, the incidence of chronic kidney function decline in patients (12.5% vs. 12.2%), and the average decline in eGFR from baseline (14.6% vs. 15%, *p* = 0.28) were not significantly different between CAR-T recipients and HSCT recipients [111]. Additionally, the strongest risk factor for developing chronic kidney function decline was the development of AKI within the first 30days post-CAR-T infusion and there is a trend towards higher rates of AKI and chronic kidney function decline amongst patients with severe CRS [111]. Available literature points to the recovery of AKI approximately 30 days after onset [109]. 

Cardiac toxicities have been described in the context of lymphoid malignancies [112]. Troponin elevation has been observed in up to 54% of patients, most notably in patients with grade ≥ 2 CRS. Moreover, one-third of patients receiving CAR-T therapy had a reduction in left ventricular ejection fraction and in long-term follow-up, 12% of patients had cardiovascular (CV) events, including decompensated heart failure, new-onset arrhythmia, and CV-related deaths. Studies have suggested that troponin, brain natriuretic peptide and cardiac strain might service as indicators for CV toxicity, and it has been proposed that tocilizumab might serve a cardioprotective role [112,113].

Long-term neurocognitive effects in patients experiencing neurotoxicity have yet to be established and are currently being studied. Few studies have reported long-term neurological outcomes after CAR-T therapy and ICANS. One study reported that at a median follow-up of 28 months, amongst a cohort of 86 patients, 10% had new neurologic findings (including cerebrovascular accidents and transient ischemic attacks), peripheral neuropathy and Alzheimer’s were found in 2 patients with CR, and 9% had depression and anxiety requiring therapy [91]. Ongoing memory impairment and unresolved neurological impairment 18 days post-infusion have been reported in 4–5% of patients [5,93]. Young age, preexisting anxiety and depression, and acute neurotoxicity were associated with worse outcomes, suggesting long term survivors of CAR-T therapy and especially those with acute neurotoxicity may benefit from mental health service follow up after completing CAR-T therapy [114]. 

While data exists for in-hospital mortality, ICU mortality (8.6%) [74], and 90-day mortality (22.4%) [115], there is a paucity of data on long-term outcomes of patients admitted to the ICU for CAR-T-related toxicity management.

## 8. Resource Utility in CAR-T Therapy Management

In addition to efficacy and toxicity, resource utilization may also be an important consideration. Early studies on cost-effectiveness assessed CAR-T therapy as cost-effective based on improved survival and quality life years gained [116,117]. Beyond the initial cost of acquisition, the main contributor to overall cost is the resource-utilization related length of inpatient hospital and ICU care [118]. Both are directly related to the rate and severity of CRS and neurotoxicity [119,120,121,122]. Certain CAR products (axi-cel compared to tisa-cel) have significantly higher resource utilization, with treatment initiated in the inpatient setting, longer hospital length of stay (19 days vs. 16 days), and higher rates of ICU admission (38% vs. 5%) [19]. Despite the higher incidence of severe toxicities, the outcomes in this population are favorable and whether observation in the ICU is required for all patients is an area that requires further investigation [74,118]. 

The feasibility of outpatient infusion and follow-up has been shown in several clinical trials and the real-world “standard of care” [123]. The promise of lower utilization of health care resources makes outpatient treatment desirable. In addition to the intrinsic risk of early and severe CRS/ICANs related to the CAR-T product, the feasibility of outpatient treatment depends on several patient and institutional factors: (1) patient-specific factors associated with risk of early and severe adverse events such as high tumor burden (elevated LDH), elevated inflammatory markers or low Eastern Cooperative Oncology Group performance status. Improved predictive models to assess individual patient risk for severe CRS or ICANS are needed to help with this decision making. (2) The necessary amount of social and family support for around-the-clock monitoring, and the willingness and capability to seek immediate medical attention at the first sign of adverse effects. (3) A health system ready to coordinate CAR-T-specific care 24/7 including trained on-call and emergency physicians and well-coordinated, frequent follow-up visits.

## 9. Future Directions

Since it’s commercialization in 2017, CAR products have shown promising early post-CAR responses and longitudinal data are slowly becoming available. To date, we have not identified robust predictors of response durability and relapse, and predictors to identify patients who are likely to achieve lasting responses versus those with early remission. Among patients with an initial response to therapy, antigen-escape—the partial or complete loss of the CAR-T target antigen—is emerging as a key mechanism of disease relapse. Antigen escape has been described in up to 28% of patients with B-cell lymphoma, up to 68% of ALL patients, and has recently also been described in multiple myeloma [124,125,126,127]. CAR-T products targeting multiple cancer antigens may overcome antigen-escape and are currently being investigated [128]. Beyond CAR-T design, nuances in the manufacturing process such as faster turnaround time and variable T-cell composition have been shown to impact CAR-T cell expansion, persistence, and efficacy [129]. 

An outstanding question that remains in the field is the role of consolidative HSCT post-CAR-T therapy. Pediatric data demonstrated decreased relapse in patients consolidation with HSCT after CD19 CAR treatment [130,131]. In contrast, adult patients receiving post-CAR consolidative HSCT did not show a benefit in event-free-survival or OS [87,132]. The optimal strategy to predict and manage post-CAR relapse, including the role of consolidative HSCT remains to be defined. 

As the early use of steroids and other interventions such as anakinra, previously considered “rescue therapies”, are administered more routinely, well-designed studies are needed to evaluate not only the short term-effect on CAR-T toxicity, but also the long-term impact of these immune suppressive strategies. Moreover, current literature focuses on relapse/complete remission/durable response, and CAR-T persistence. Robust studies are evaluating patient-centered outcomes, such as functional status, disposition (home, nursing home hospice, etc.) and continued need for organ support (e.g., chronic ventilatory support and renal replacement therapy), in patients with grade ≥ 2 toxicity requiring ICU who are currently lacking. care.

Finally, CAR-T therapy has been studied in early phase trials of solid tumors, such as neurobloastoma, sarcoma, non-small cell lung cancer and renal cell carcinoma [133,134,135]. Some of the hurdles for CAR-T therapy in solid tumors have been noted to be a lack of tumor-specific antigens, inefficient CAR-T cell trafficking into tumor sites, toxicity and antigen escape. On-target/off-tumor toxicity is a major obstacle to CAR-T therapy in solid tumors. In contrast to CD19 and BCMA, which are highly specific B-cell antigens, solid tumor-associated antigens are overexpressed in solid tumors and on healthy tissue leading to reports of fatal pulmonary toxicity and encephalitis in early stage trials [136,137]. Although the clinical success of CAR-T cell therapy in solid tumors has not paralleled that of hematologic malignancies yet, some clinical responses provide encouragement for further trials.

The future holds the promise that novel CAR-T constructs, and novel cell products, such as CAR- natural killer cells (NK) or CAR-macrophages can target a wide range of hematologic and solid-tumor malignancies. CAR-NK cells have demonstrated a response rate of 73% and a complete response of 63% in patients with R/R non-hodgkins lymphoma or CLL. CAR-NK has several advantages over CAR-T cells [138]; is not associated with an inflammatory surge of cytokines; and thus presents a lower risk of CRS and ICANS [139], reduced risk of allo-reactivity, and can be manufactured “off-the-shelf” from existing NK-cell lines, cord blood, and induced-pluripotent stem cells. In contrast to T and NK cells, macrophages account for the majority of infiltrating immune cells in solid tumors. CAR-macrophages are, therefore, a promising mechanism currently under investigation to overcome the anti-inflammatory tumor environment of solid-tumors [139,140].

In summary, CAR-T cell therapies have revolutionized cancer treatment and have demonstrated excellent response rates in relapsed and refractory malignancies. Although the potential for severe toxicities exists, standardized monitoring has enabled early recognition and intervention, leading to the successful translation of CAR therapy from clinical trials to standard-of-care practice. Ongoing improvements in toxicity mitigation strategies, CAR product design, manufacturing, and patient selection, as well as the identification of novel cancer-specific targets, will further expand the application of CAR-cell therapies for a growing number of hematologic and solid-tumor malignancies. However, with more patients being successfully treated, long-term follow-up and management of the potential long-term effects of CAR-cell therapy remain to be investigated.

## Figures and Tables

**Table 1:** Pivotal licensing trials for FDA-approved CAR-T products.

CAR-T Product	Antigen Target	Co-Stimulatory Domain	Indication	CR	CRS Any Grade	CRS Grade ≥ 3	ICANS AnyGrade	ICANS Grade ≥ 3
axicabtagene ciloleucel (YESCARTA^®^) [5]	CD19	CD28	DLBCL Mediastinal B-cell lymphomaFollicular lymphoma	54%	85%	13%	64%	28%
tisagenlecleucel (KYMRIAH^®^) [13]	CD19	4-1BB	DLBCL ALL	40%	58%	22%	21%	12%
lisocabtagene maraleucel (BREYANZI^®^) [12]	CD19	4-1BB	DLBCL High-grade B-cell lymphoma Mediastinal B-cell lymphomaFollicular lymphoma	53%	42%	2%	30%	10%
brexucabtagene autoleucel (TECARTUS^®^) [11]	CD19	CD28	Mantle cell lymphoma ALL	67%	91%	15%	63%	31%
idecabtagene vicleucel (ABECMA^®^) [14]	BCMA	4-1BB	Multiple myeloma	33%	85%	5%	18%	3%
ciltacabtagene autoleucel (CARVYKTI^®^) [15]	BCMA	4-1BB	Multiple myeloma	67%	51%	4%	21%	9%

Abbreviations: ALL: Acute lymphoblastic leukemia; BCMA: B-Cell maturation antigen; CAR-T: Chimeric Antigen Receptor-T; CR: Complete response; CRS: Cytokine release syndrome; DLBCL; Diffuse large B-cell lymphoma; ICANS: Immune effector cell-Associated Neurotoxicity Syndrome (ICANS).

**Table 2:** Real World Experience.

Study (First Author, Year)	Product	Number of Patients Infused	ORR/CR (%)	CRS (%) Any/Gr 3+	ICANS (%) Any/Gr 3+
Jacobson, 2022 [16]	Axi-cel	1297	73/56	83/8	55/24
Kwon, 2023 [17]	Axi-cel	134	60/42	88/8	42/18
	Tisa-cel	127	54/34	73/6	16/5
Kuhnl, 2022 [21]	Axi-cel	224	77/52	93/8	37/16
	Tisa-cel	76	57/44	56/8	15/4
Bachy, 2022 [22]	Axi-cel	213	80/60	86/5	48/14
	Tisa-cel	419	66/42	75/9	22/3
Riedell, 2022 [19]	Axi-cel	156	52/44	85/9	56/39
	Tisa-cel	84	41/35	39/1	11/1
Bethge, 2022 [23]	Axi-cel	173	74/42	81/10	44/16
	Tisa-cel	183	53/32	65/13	22/7
Gauthier, 2022 [20]	Axi-cel	68	75/43	87/7	62/29
	Tisa-cel	31	58/32	70/0	23/13
Jacobson, 2020 [24]	Axi-cel	122	70/50	93/16	70/35
Iacoboni, 2021 [25]	Tisa-cel	75	60/32	71/5	15/1
Nastoupil, 2020 [26]	Axi-cel	275	82/64	91/7	69/31
Pasquini, 2019 [31]	Axi-cel	533	74/54	83/9	53/17
Iacoboni, 2022 [28]	Brexu-cel	33	91/79	91/3	64/36
Wang, 2023 [29]	Brexu-cel	168	90/82	90/8	61/32
Hansen, 2023 [30]	Ide-cel	108	64/34	82/4	15/5

Abbreviations: ORR; overall response rate; CR: complete response; CRS: cytokine release syndrome; ICANS: Immune-Effector-Cell-Associated Neurotoxicity Syndrome (ICANS), Gr: grade.

**Table 3:** CRS Grading and Management.

	**Management**
**G1:** **Fever: ≥38 °C without hypotension or hypoxia**	Supportive care Empiric antibiotics
**G2:** **Fever: ≥38 °C** **AND** **Hypotension: not requiring vasopressors** ***And/or*** **Hypoxia: Oxygen ≤6 L/min**	Supplemental oxygen IV fluid bolus Tocilizumab 8 mg/kg IV, repeat q8h if no improvement, max 3 doses Dexamethasone 10 mg IV (or equivalent) every 12 h if hypotension persists after 2 fluid boluses and 1–2 doses of tocilizumab Manage per G3 if there is no improvement after 24 h
**G3:** **Fever: ≥38 °C** **AND** **Hypotension: requiring vasopressors** ***And/or*** **Hypoxia: requiring high-flow oxygen, face mask, nonrebreather mask**	Admit patient to ICU Tocilizumab as per G2 if maximum dose is not reached within 24 h period dexamethasone 10 mg IV every 6 h (or equivalent); taper once improved to G1
**G4:** **Fever: ≥38 °C** **AND** **Hypotension: requiring multiple vasopressors** ***And/or*** **Hypoxia: requiring positive pressure ventilation (CPAP, BiPAP, mechanical ventilation)**	Tocilizumab as per G2 if maximum is not reached within 24 h Consider anakinra, siltuximab, ruxolitinib High-dose methylprednisolone If not improving, consider methylprednisolone 1000 mg IV 2 times a day or alternate/rescue therapy

Abbreviations; BiPAP = bilevel positive airway pressure; CPAP = continuous positive airway pressure; G1: grade 1; G2: grade 2; G3: grade 3; G4: grade 4; ICU: intensive care unit; IV: intravenous. Adopted from NCCN Guidelines.

**Table 4:** Diagnostic criteria HLH and CAR-HLH.

**Criteria**	**HLH 2004 Criteria**	**H-Score**	**CAR-T HLH/MAS**
**Fever**	Fever > 38.4	Fever > 38.4	N/A
**Organ assessment**	Splenomegaly	Organomegaly ‑ Splenomegaly ‑ Hepatomegaly ‑ Or both	≥2 organ systems with grade ≥ 3 organ failure * after CRS ‑ liver ‑ renal ‑ pulmonary
**Cytopenias (≥2 of 3 lines)**	Hemoglobin < 90 g/L Platelets < 100 × 10^9^/L Neutrophils < 1.0 × 10^9^/L	Hemoglobin < 9.2 g/dL Platelets < 110,000/mm^3^ Leukocytes < 50,000/mm^3^	N/A
**Triglycerides**	≥3.0 mmol/L	>1.5 mmol/L	N/A
**Fibrinogen**	≤1.5 g/L	≤2.5 g/L	
**Ferritin**	Ferritin ≥ 500 ng/mL	Ferritin > 2000 ng/mL	Ferritin > 10,000 ng/mL (during CRS)
**Additional laboratory considerations**	Low or absent NK-cell activity (according to local laboratory reference)	Serum GOT ≥ 30 IU/L	
	Soluble CD25 (i.e., soluble IL-2 receptor) ≥ 2400 U/mL		
**Histopathology**	Hemophagocytosis	Hemophagocytosis	Hemophagocytosis on histopathology or CD68 immunohistochemistry

Abbreviations: CAR-T: Chimeric Antigen Receptor-T; CRS: Cytokine release syndrome; HLH: hemophagocytic lymphohistiocytosis; IL: Interleukin; MAS: Macrophage Activation Syndrome; NK: Natural killer. * Grading as per Common Terminology Criteria for Adverse Events, U.S. Department of Health & Human Services. *Common Terminology Criteria for Adverse Events (CTCAE) Version 4.0* https://evs.nci.nih.gov/ftp1/CTCAE/CTCAE_4.03_2010-06-14_QuickReference_5x7.pdf (2010) (accessed on 1 May 2023).

**Table 5:** ICANS grade and management.

**Grading**	**No Concurrent CRS**	**Additional Therapy if Concurrent CRS**
**G1:** **ICE score: 7–9** **Normal consciousness**	Supportive care ^a^	Tocilizumab 8 mg/kg IV Dexamethasone 10 mg IV if more than 1 dose of tocilizumab is required for ongoing CRS
**G2:** **ICE score: 3–6** ***And/or*** **Mild somnolence awaking to voice**	Dexamethasone 10 mg IV and reassess Repeat every 6–12 h if no improvement. Taper once symptoms improve to G1	Consider ICU if CRS and ICANS ≥ 2 Tocilizumab per grade 1
**G3:** **ICE score: 0–2** ***And/or*** **Awakening only to tactile stimulus** ***And/or*** **Any clinical seizure or non-convulsive seizures on EEG that resolve with intervention** ***And/or*** **Focal edema on neuroimaging**	Consider transfer to ICU Dexamethasone 10 mg IV every 6–12 h or methylprednisolone equivalent (1 mg/kg IV every 12 h). Consider repeat imaging if persistent ≥ G3	Tocilizumab as per grade 1
**G4:** **ICE score: 0** *And/or* **Stupor or coma** *And/or* **Prolonged seizure (** **>** **5 min) or** **repetitive clinical or electrical seizures without return to baseline** *And/or* **Diffuse cerebral edema on neuroimaging, clinical signs of elevated intracranial pressure (decelerate posturing, papilledema, Cushing’s triad)**	Admit patient to ICU Consider mechanical ventilation for airway protection High-dose methylprednisolone IV 1000 mg once a day, up to 3 times per day if refractory Consider anakinra and/or additional rescue therapy for persistent or worsening symptoms ^b^ Repeat CNS imaging Treat status epilepticus per institutional guidelines	Tocilizumab as per grade 1
ICE score: Orientation: orientation to year, month, city, hospital: 4 points Naming: ability to name 3 objects (e.g., point to clock, pen, button): 3 points Following commands: ability to follow simple commands (e.g., “Show me 2 fingers” or “Close your eyes and stick out your tongue”): 1 point Writing: ability to write a standard sentence (e.g., “Our national bird is the bald eagle”): 1 point Attention: ability to count backward from 100 by 10: 1 point 0 points if patient is unresponsive or unable to perform		

^a^ For products associated with more neurotoxicity (axicabtagene–ciloleucel or brexucabtagene autoleucel), administration of steroids starting at G1 ICANS and use of high-dose steroids at G3 may be considered. ^b^ Rescue therapies: anakinra, siltuximab, ruxolitinib, cyclophosphamide, antithymocyte globulin, or intrathecal hydrocortisone (50 mg) plus methotrexate (12 mg). Abbreviations: CRS: cytokine release syndrome; EEG = electroencephalogram; G1: grade 1; G2: grade 2; G3: grade 3; G4: grade 4; ICE = Immune-Effector-Cell-Associated Encephalopathy; ICP = Intracranial Pressure; ICU: intensive care unit; IV: intravenous.
